# Direct visualization of radiation-induced transformations at alkali halide–air interfaces

**DOI:** 10.1038/s42004-021-00486-2

**Published:** 2021-04-08

**Authors:** Shawn L. Riechers, Nikolay G. Petrik, John S. Loring, Mark E. Bowden, John B. Cliff, Mark K. Murphy, Carolyn I. Pearce, Greg A. Kimmel, Kevin M. Rosso

**Affiliations:** Pacific Northwest National Laboratory, Richland, WA USA

**Keywords:** Nuclear chemistry, Scanning probe microscopy, Characterization and analytical techniques, Atmospheric chemistry

## Abstract

Radiation driven reactions at mineral/air interfaces are important to the chemistry of the atmosphere, but experimental constraints (e.g. simultaneous irradiation, in situ observation, and environmental control) leave process understanding incomplete. Using a custom atomic force microscope equipped with an integrated X-ray source, transformation of potassium bromide surfaces to potassium nitrate by air radiolysis species was followed directly in situ at the nanoscale. Radiolysis initiates dynamic step edge dissolution, surface composition evolution, and ultimately nucleation and heteroepitaxial growth of potassium nitrate crystallites mediated by surface diffusion at rates controlled by adsorbed water. In contrast to in situ electron microscopy and synchrotron-based imaging techniques where high radiation doses are intrinsic, our approach illustrates the value of decoupling irradiation and the basis of observation.

## Abstract (web summary)

Halide salts exhibit complex radiation-induced reactions that are relevant in atmospheric chemistry, but detailed characterizations in high-level radiation fields are challenging to obtain. Here, the authors use a custom atomic force microscope to study alkali halide surface transformations in situ under 18 kGy/hr irradiation.

## Introduction

Reactions induced by ionizing radiation at material interfaces are important in many fields including atmospheric chemistry^1–3^, nuclear reactor material design, radioactive waste repositories^4–6^, dosimetry^7^, medical devices^8,9^, and space science engineering^10^. Fundamental understanding of these systems often benefits from direct detailed observations under in situ irradiation conditions in real-time to be able to infer impacts over larger spatial and temporal scales^9,11^. The lack of such data is due to the difficulty in simultaneously providing environmental control and detailed characterization in a high-level radiation field. The rise of novel in situ holders for electron microscopy, and combined synchrotron-based microscopy and spectroscopy have allowed for significant progress in this regard^12–17^. However, persistent challenges include the fact that in most cases the radiation environment and timescales achieved usually do not represent that of the reaction of interest, and that the radiation being used is often that necessary to make the measurement itself^14–16^. New techniques in which the radiation environment is flexible and decoupled from the measurement probe, particularly at high resolution, would represent a new experimental paradigm.

Halide salts exhibit complex radiation-induced reactions, particularly in contact with ambient air where nitrogen and water undergo radiolysis. This is particularly important for understanding the impact of sea salt particles (NaCl, bromides, and iodides) on the chemistry of Earth’s atmosphere, such as chlorine balance, ozone levels, etc^11,18–20^. Earth’s upper atmosphere is constantly irradiated by cosmic rays and energetic UV photons^1–3^ but it is poorly understood how this catalyzes reactions in the lower atmosphere^20,21^ and to what extent this involves air/particle interfaces.

Radiolysis and UV photolysis of air produces a wide range of short-lived, highly reactive intermediate species, ions, and radicals^22–29^. When an alkali halide salt is irradiated in air, nitrate of the alkali metal forms on the surface as shown by infrared (IR) spectroscopy, X-ray diffraction (XRD), and chemical analysis^21,30–32^. The most important radiolytic precursor for the NO_3_^−^ ion is NO_2_ that is produced in air and subsequently reacts with the halide^19,20,30–33^:

1 $${\mathrm{2NO}}_{2\left( {\mathrm{g}} \right)} + {\mathrm{MeX}}_{\left( {\mathrm{s}} \right)} \leftrightarrow \left( {{\mathrm{NO}}^ + + {\mathrm{NO}}_3^ - } \right)_{\left( {{\mathrm{ads}}} \right)}\,\\ + {\mathrm{MeX}}_{\left( {\mathrm{s}} \right)} \to {\mathrm{NOX}}_{\left( {\mathrm{g}} \right)} + {\mathrm{MeNO}}_{3\left( {\mathrm{s}} \right)}$$

where Me = alkali metal and X = halide.

This reaction is efficient, and hundreds of monolayer equivalents of alkali halides are converted into alkali nitrates due to desorption of the halogen from the lattice into the gas phase in the form of volatile nitrosyl halide. Sub-micron-sized crystallites on the surface of alkali halide crystals, attributed to these radiation-induced nitrates, have been studied using electron microscopy^34^. However, the evolution of the surface is complex, involving adsorption/desorption, dissolution, migration, and finally nucleation and growth of the nitrate phase. In such cases, ex situ imaging, while informative and valuable^35^ makes it more difficult to link material sources to sinks as the surface evolves. Understanding these complex processes would greatly benefit from developing the ability to visualize the mass transport pathways (i.e., dissolution, nucleation, and growth) directly in situ at the nanoscale.

Here, we achieve this goal using a novel atomic force microscope (AFM) designed for this purpose, and present new insights into the details of alkali halide surface transformation under controlled irradiation conditions. Using our “radAFM”, we examined potassium bromide (KBr) (100) surfaces reacting in air and air/argon mixtures under controlled relative humidity (RH), with ionizing radiation directed at the interface from underneath the sample using a compact X-ray source (Emax = 20 keV). The surface structural evolution was monitored with nanometer resolution in situ under 18 kGy/h irradiation, using a combination of topography, phase, and amplitude imaging to follow dissolving step edges, evolving surface composition, and the nucleation and growth of individual KNO_3_ crystallites. A variety of ex situ techniques were also used to characterize the surface reactions.

## Results

### X-ray induced potassium nitrate growth

**Fig. 1:**
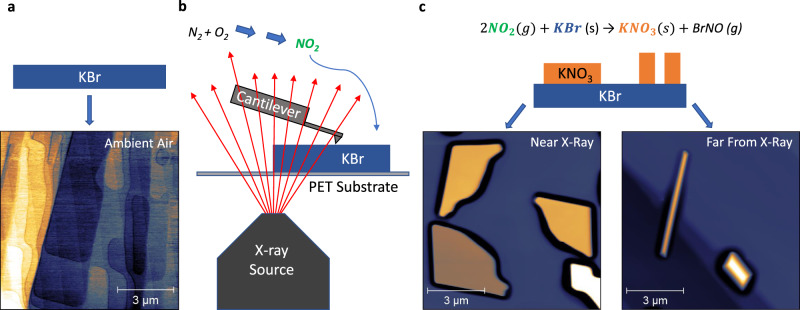
Schematic of the KBr irradiation setup and proposed X-ray-induced reaction pathway. Freshly cleaved KBr (~3 × 3 × 1 mm) (**a**) is irradiated in a controlled atmosphere (**b**). X-rays penetrate a 0.25 mm polyethylene terephthalate (PET) substrate and react with air in a sealed reaction cell. Radiolitic products are generated, including NO_2_, which react with the KBr substrate and result in the growth of KNO_3_ crystals. After irradiation with 400 kGy in an Ar/air mixture, the resulting volume and morphology of KNO_3_ growth varies by proximity to the X-ray source (**c**).

**Fig. 2:**
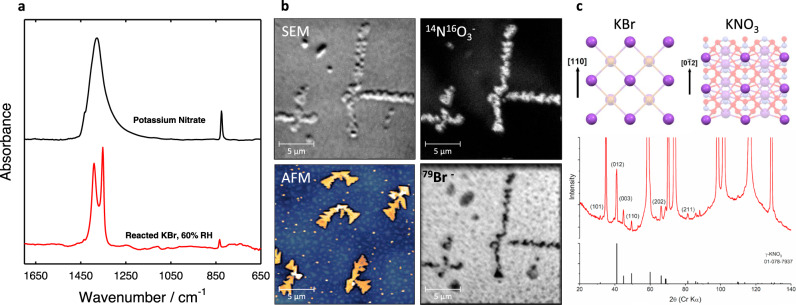
Analysis of KBr crystals after irradiation confirm the presence of γ-KNO_3_. IR absorption bands at 1391, 1352, and 833 cm^−1^ of the irradiated KBr were consistent with KNO_3_ powder standards and reported stretching and bending vibrational modes of KNO_3_ crystallites^34,44^. IR bands characteristic of individual NO_3_^−^ ions, produced on X-ray and gamma-ray irradiated alkali halide surfaces in air^21,22,30,34^, were also present (**a**). Nano-SIMS analysis of an irradiated KBr sample confirmed crystallites contain higher concentrations of NO_3_^−^ ions, and lower concentrations of Br^−^ ions (**b**). The µXRD pattern for the irradiated sample exhibited intense peaks corresponding to the KBr substrate and weaker peaks corresponding to surface growths, which were identified as the trigonal, (also gamma or phase III) form of KNO_3_ (**c**). The lattice vectors marked in (**c**) are their projections in the plane of the figure.

Prior to irradiation, freshly cleaved KBr (100) surfaces are well-ordered with large atomically-flat terraces truncated by linear to curvilinear steps (Fig. S1). This initial topography turned out to be metastable in ambient air, as sharp intersections of steps became rounded after ~1 h, likely due to water adsorption and consequential mild surface reconfiguration (Fig. 1a and Fig. S1). This surface characterization decoupled from irradiation as well as the effect of heating to 50 °C overnight (Fig. S2), provide a baseline understanding of the KBr surface devoid of irradiation. Our radAFM sample cell is configured to control the local gas/liquid environment during irradiation (see Supplementary methods). To first explore the effects of an extreme total dose, samples such as these were irradiated with 20 keV X-rays inside the radAFM cell after 1 h of equilibration in a given gas/humidity, and then imaged (Fig. 1b). A typical dose rate in the cell was 18 kGy/h as measured with GafChromic dosimetry films with total doses from 120 to 230 kGy. As a point of reference, a typical dose rate for TEM is 5 × 10^6^ kGy/h^36^, for X-Ray reflection interface microscopy is 10^4^ kGy/h^12^, and experienced by the international space station is 10^−8^ kGy/h^37^, while total doses expected for high-level nuclear waste repository materials over 10,000 years is 2 × 10^6^ kGy^38^, and required for medical device sterilization is 25 kGy^39^. After irradiation new island-like euhedral crystallites consistent with KNO_3_ had formed on the surface, bearing regular orientations consistent with heteroepitaxial growth (Fig. 1c). The crystallite morphology included triangular, similar to previous ex situ reports^34^, but also rhombic, needle-like, and branched structures (Figs. 1c, S3). The morphology observed for a given condition expressed a dependence on position relative to the X-ray source, Table S1. Unexpectedly, post-mortem characterization of this product phase showed it to be the γ KNO_3_ polymorph, a ferroelectric material, using ex situ IR spectroscopy (Fig. 2a), NanoSIMS (Fig. 2b), and µXRD (Fig. 2c), see Supplementary Methods KBr/KNO_3_ analysis. A Pawley fit of the γ KNO_3_ XRD peaks resulted in lattice parameters of *a* = 546.6(2) nm and *c* = 900.8(4) nm which are within experimental error of the reported values for the bulk compound^40^. This indicates that although the γ KNO_3_ crystals are epitaxially aligned to KBr, they are not coherently strained to the substrate since the lattice mismatch is −7% and +17% in orthogonal directions along the substrate surface. This is discussed further in the SI and Fig. S4. As far as we are aware, this polymorph does not form under ambient conditions^41,42^, suggesting the possible importance of heteroepitaxial strain and/or surface electric fields in determining its structure^42,43^. On the KBr surface, the ferroelectric properties of these product crystallites were readily evident by AFM using piezoresponse force microscopy (PFM), and magnetic force microscopy (MFM) which revealed significant magnetic susceptibility consistent with γ KNO_3_ (Fig. S5) albeit previously documented only for crystallites less than 30 nm in diameter^43,44^. Hence the radiation-induced chemistry examined here appears to provide a novel pathway to γ KNO_3_.

With increasing distance from the center of irradiation, surface coverage and crystallite size decreased, and crystallite morphology changed (Figs. 1c, S3). We believe this effect is primarily due to a decreasing rate of production, and therefore available concentration, of radiolytically derived species as a function of lateral distance from the X-ray source. To begin to understand the radiation-induced source-to-sink mass transfer process, we measured the total volume of KNO_3_ crystallites on the KBr surface using AFM topography (Fig. 1c) and determined the average number of KNO_3_ formula units per square centimeter was 8.6 × 10^15^. This agrees remarkably well with the coverage calculated under these conditions based on the expected amount of nitrates produced due to air radiolysis: Given the known radiation-chemical yield of NO_2_ measured during gamma irradiation of air^45,46^ our X-ray dosimetry, and the stoichiometry of Reaction (1), 3.2 × 10^16^ KNO_3_ molecules/cm^2^ should be produced. Both estimates confirm that ~10–100 monolayers of KBr were converted into KNO_3_ crystallites, which supports the proposed mechanism of radiation-induced nitration of the KBr surface.

Our ex situ studies also enabled us to assess the influence of gas-phase composition on the surface nitration rate, which we explored by controlling humidity and by diluting the air concentration with Ar. The critical role of adsorbed water as a medium for mass transfer was particularly evident. For example, KBr irradiated in air under humid conditions resulted in a greater amount of KNO_3_ surface coverage than in ambient or dry conditions, likely due to an increase in ion surface mobility under humid conditions^47^. Also, we discovered that the KNO_3_ surface coverage did not change significantly when irradiated in air or in a 99% Ar/1% air mixture, under dry or humid conditions. This can be attributed to participation of the Ar gas background as a sensitizer for radiolysis, where rare gas ions enable efficient transfer of charge/excitation energy to the molecules of interest^22,46,48^.

### In situ observation of potassium nitrate nucleation and growth

**Fig. 3:**
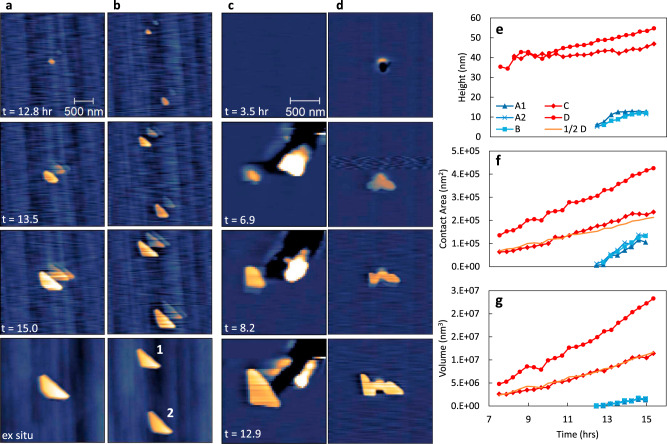
Time-lapse analysis of X-ray irradiation-induced KNO_3_ growth on KBr measured by in situ AFM topography. KBr single crystals were equilibrated for 1 h in 0.1% RH Air (**a**, **b**) and 60% RH 99% Ar/1% air mix (**c**, **d**) prior to irradiation at 18 kGy/h. Growth over time of individual KNO_3_ crystals shown in (**a**–**d**) and ½ the value of (**d**) for comparison is measured by height (**e**), contact area (**f**), and volume (**g**). It should be noted that in the dry case the AFM tip shape changed over time, creating a multi-tip artifact. After in situ imaging the tip was replaced to capture the artifact free morphology, a-b last frame.

In situ AFM imaging during irradiation was then performed to monitor the details of KBr surface dissolution coupled to KNO_3_ nucleation and growth. Exclusion of water provided a reference case for understanding the transformation rate arising primarily from radiolytic NO_2_ production alone. This limits the surface transport rate of K^+^ ions and mono-ions of NO_3_^−^, which have been shown to first form NO_3_^−^ clusters en route to crystalline KNO_3_^30–33,48,49^. We observed that irradiation in dry air produced the lowest coverage of KNO_3_ crystallites, and nucleation was first observed only in regions of high step edge density, rich in defect sites, after a relatively high dose of ~230 kGy or 12.9 h of irradiation (Fig. 3a, b and Supplementary Movie S1–3). Initial nuclei were anhedral round particles but quickly grew to adopt an obtuse trigonal shape. Analysis of particle growth over time for particles A, B1, and B2 revealed an average 2.7 nm/h increase in height, a surface contact area growth rate of 5.1 × 10^4^ nm^2^/h, and a total growth rate of 6.7 × 10^5^ nm^3^/h (Fig. 3e–g, blue traces). This constitutes ~8.6 × 10^6^ KNO_3_ molecules attaching to each crystallite per hour.

In contrast, in elevated humidity the KNO_3_ formation rate was much faster. Irradiation of the KBr samples in an atmosphere of 99% Ar and 1% air with 60% relative humidity (RH) resulted in nucleation after 124 kGy or 6.9 h of irradiation, which is nearly half the dose required in the absence of moisture. Nucleation of some particles again appeared to be initiated by defect sites in the surface (Fig. 3c, d, Supplementary Movie S4–6). Growth rates in-plane and normal to the surface clearly showed a dependence on adsorbed water. For example, the vertical growth rate of some particles was as low as for the dry air conditions, whereas the lateral growth was often enhanced (Fig. 3e–g). Under these humid conditions, the average height, lateral growth, and total growth rate of a single crystal were 1.8 nm/h, 2.2 × 10^4^ nm^2^/h, and 1.3 × 10^6^ nm^3^/h, respectively, constituting an overall KNO_3_ attachment rate of 1.5 × 10^7^ molecules/h. Thus, the KNO_3_ attachment rate was ~74% faster in humid Ar/air, than in dry air, in agreement with ex situ results.

KNO_3_ crystal growth resulting from irradiation was found to be similar despite several differences in the cell configuration under ex situ (quartz cover) and in situ conditions (slightly reduced volume, silicon nitride tip, PEEK tip holder, and Viton bellows above sample). This suggests that if there are gas-phase chemical species generated from these materials during irradiation, they have little interaction with the salt surface.

### Dynamics at the interface

**Fig. 4:**
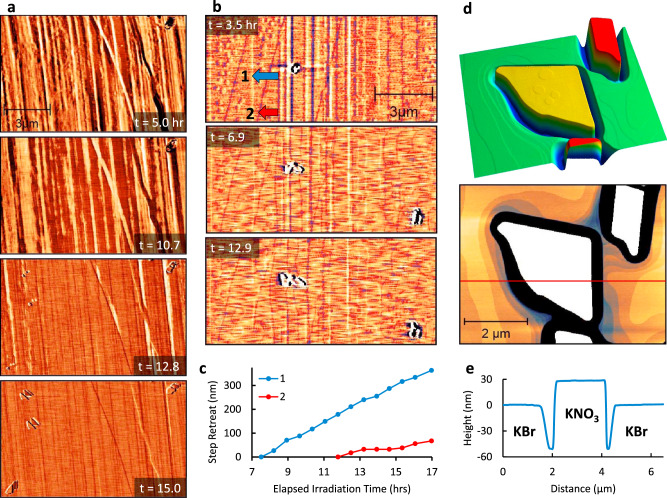
Surface evolution of irradiated KBr observed by AFM phase and amplitude. Time-lapse AFM tapping mode phase images of X-ray irradiation-induced KNO_3_ growth on KBr, after 1 h of exposure to 0.1% RH Air. A transition in composition at the interface of the dry substrate is observed before nucleation begins (**a**). Time-lapse amplitude images of KBr irradiated after 1 h of exposure to 60% RH argon/air mixture reveal step edge dissolution (**b**). Measurements of local step edge retreat rates (**c**), indicated by the base of arrows 1–2 in (**b**), reveal an increase in dissolution near KNO_3_ crystallites giving rise to bowed step edges. Over time this increased local dissolution rate gives rise to depressed regions at the perimeter of KNO_3_ crystals, such as those shown by 3D and 2D topography projections (**d**), and cursor profile (**e**) after long-term ex situ irradiation.

To better understand KBr dissolution and the production of K^+^ source material for KNO_3_, we exploited the ability of AFM to detect important physicochemical changes in material properties. During KBr irradiation in dry air, phase contrast data, an imaging channel that is sensitive to surface hardness or viscoelasticity and tip/surface adhesion^49–51^, revealed a consistent phase shift over the course of ~7 h (Fig. 4a, Supplementary Movie S3). A positive phase shift (bright contrast), initially ubiquitous on the KBr surface, gave way to a negative shift (darker contrast, from 5.7 to 0.9 degrees lower) for the remainder of the experiment. The onset of KNO_3_ crystal nucleation was not observed until after this surface transformation was complete. This change in the surface property was not observed for KBr irradiated in humid Ar/air (Supplementary Movie S6). While phase contrast can be attributed to many factors, we hypothesize the phase shift, coincident with the underlying topography and nucleation, observed under dry conditions may be attributed to the accumulation of charged nitrate species on the surface, which would affect the tip/surface interaction and thus phase contrast, until a concentration threshold is reached and a phase contrast that is similar to the hydrated condition is observed and nucleation is possible.

### Potassium bromide dissolution

Dissolution rates at step edges were quantified and compared directly with KNO_3_ product growth rates. In particular, in situ imaging of individual KBr step edges, prominently detectable in the amplitude channel, allowed their retreat to be clearly observed (Fig. 4b, Supplementary Movie S5). In addition to step edge dissolution, occasionally dissolution on top of terraces is also observed as an atomic step depression that grows over time. These disk-like features seen on the KBr substrate are distinct from the disk-like features of growth on larger KNO_3_ crystallites (Fig. 4b, c). Due to the absence of these disk-like dissolution features on KBr terraces after longer ex situ irradiation experiments it is unlikely that these are a direct effect of irradiation damage. Dissolution is likely associated with the reaction of the undercoordinated KBr sites with adsorbing NO_2_ molecules to produce mobile KNO_3_ species. Freshly cleaved unirradiated KBr surfaces observed over the same timeframe did not show any dissolution (Fig. S1). Upon nucleation of KNO_3_ the retreat rate of steps nearest the crystallites increases, as shown by arrows 1 and 2, resulting in a “bowed” region of mass transfer consumption around crystallites. These initial stages of nucleation appeared to dictate the morphology of larger KNO_3_ crystals. As the KNO_3_ crystallites reach a certain size, trenches formed in the surrounding KBr that are deeper than the crystallites are high, as shown by 3D and 2D topography (Fig. 4d, e). This dissolution process is fundamentally distinct from dissolution in water or irradiation-induced desorption under vacuum^51,52^. By measuring step edge retreat rates, the rate of dissolution was compared with the rate of crystallite growth. The average step retreat rates in the region shown in Fig. 4b constitute 1.0 × 10^5^ KNO_3_ molecules released per hour in one square µm of the KBr surface. The rate of KNO_3_ crystallite growth for this same region requires that 1.5 × 10^7^ KNO_3_ are incorporated per hour. Therefore, the K^+^ required for the growth of a single KNO_3_ crystal can be accommodated by the dissolution of a circular region with a diameter of ~13.6 µm.

## Discussion

Collectively, our in situ study provides important new insight into radiation-induced transformations of salt crystal surfaces relevant to an atmospheric setting. The visualization data reveal specifically how the initial reaction between KBr and radiolytic NO_2_ occurs preferentially along individual step edges. This is followed by an accumulation of mobile KNO_3_ molecules until a threshold is surpassed for KNO_3_ crystallite nucleation. We suggest that this pre-nucleation step is sensitive to localized conditions, including humidity, the flux of radiolytic reactants to the surface, and gas-phase composition. After the onset of nucleation, γ KNO_3_ crystal growth proceeds at a steady rate as KNO_3_ molecules accumulate and are incorporated into crystallites. A linear growth rate is observed for irradiation up to 15 h, but only after a certain induction period which results in the onset of nucleation, thus the apparent kinetics are more complex. This stable growth of the new crystalline phase is less dependent on local conditions. It is likely that surface diffusion is the rate-limiting step in the growth of KNO_3_ crystals, as deep trenches in KBr around growing crystallites are indicative of a significant gradient in KBr dissolution efficiency. Such observations are likely generalizable to the complex interactions directing the interfacial chemistry of alkali halide particles in air under the influence of ionizing radiation. Prospects for future work include developing the ability to specifically differentiate the roles of atmospheric radiolytic reactants from the possible influence of radiation-induced defects at the solid surface. The ability to visualize radiation-induced processes in situ, for these systems and beyond, provides a basis for better understanding the consequences of ionizing radiation at interfaces and the development of more accurate predictive models.

## Methods

### AFM/X-ray Integration

The irradiation AFM system utilizes an Asylum MFP-3D AFM integrated with a Moxtek MAGPRO 60 kV, 12 W X-ray source. Custom radiation shielding was manufactured from ¼” Al sheets and is equipped with an interlock system to ensure safe operation of the X-ray. The maximum X-ray output of 12 W at 20 kV and 600 µA was used for these studies. The X-ray dose rate was measured by irradiating thin radiachromic films (HD-V2 GafChromic film, International Specialty Products, Wayne, NJ, USA) placed in the same configuration as samples for short durations at varying outputs. The dose response was calibrated to a set of films irradiated to a known dose using ^137^Cs (662 keV). The true dose curve (in Gy) was used to extrapolate a dose rate at maximum power (12 W) of 17.7 kGy/h within the irradiated area. Over the course of hundreds of hours of in situ X-ray irradiation of the AFM scan head no discernable degradation of optical or AFM imaging quality was observed.

Samples were placed on the sample stage directly over the X-ray source in a polyetheretherketone (PEEK) fluid cell that is modified with a clear 0.25 mm thick sheet of polyethylene terephthalate (PET) as the substrate which reduces attenuation of X-rays while providing adequate support and visual confirmation of the X-ray position. The X-ray source/sample separation was minimized to less than 3 mm and aligned on the corner of the KBr crystal such that three fourths of the beam irradiates the surrounding gas environment unhindered. A custom-built quartz cover was used to seal the cell during ex situ irradiation experiments, while Viton bellows and the AFM scan head seal the system during in situ irradiation experiments. The gas environment within the fluid cell was controlled by a custom-built mass flow control system which controls both flow rate and humidity.

### KBr/AFM imaging

KBr single crystals (10 mm × 10 mm × 50 mm, International Crystal Laboratories, NJ, USA) were cleaved to 0.3–0.5 cm^2^ immediately prior to use. AFM imaging was conducted in tapping mode using a tip with a 40 N/m cantilever (RTESPA-300, Bruker) using as small a drive amplitude as possible to minimize tip/surface interactions. Images were processed using Gwyddion (v 2.55, http://gwyddion.net/) to plane flattened and row aligned (median of differences or polynomial 4) after masking crystallites. Time-lapse videos were made by creating stacks in Image J (1.47 v, http://imagej.nih.gov/ij) and aligning by common fiducial features (plugin: NMS_fixTranslation_ver1.ijm, 2014, Nicholas M. Schneider).

## Supplementary information

Supplemental Information

Description of Additional Supplementary Files

Supplementary Movie 1

Supplementary Movie 2

Supplementary Movie 3

Supplementary Movie 4

Supplementary Movie 5

Supplementary Movie 6

## Data Availability

The main data supporting the finding of this study are available within the paper and its Supplementary Information file. Other relevant data are available from the corresponding author upon reasonable request.
