# Supplementary material for: Direct visualization of radiation-induced transformations at alkali halide–air interfaces
Source: Commun Chem. 2021 Apr 8;4:49. doi: 10.1038/s42004-021-00486-2 (PMC9814822; doi:10.1038/s42004-021-00486-2)
Supplement: Supplementary file 1 — Supplemental Information [file 42004_2021_486_MOESM1_ESM.pdf]

Supplementary Information

Direct Visualization of Radiation-Induced Transformations at Alkali Halide-Air Interfaces

Shawn L. Riechers, Nikolay G. Petrik, John S. Loring, Mark E. Bowden, John B. Cliff, Mark K. Murphy, Carolyn I. Pearce, Greg A. Kimmel, and Kevin M. Rosso

Correspondence to: shawn.riechers@pnnl.gov, kevin.rosso@pnnl.gov

Supplementary methods

In Situ Environment

The design of the MFP-3D closed fluid cell used as the environmental chamber can be found online (Oxford/Asylum, https://afm.oxinst.com/products/mfp-3d-liquid-environmentalcontrol/mfp-3d-closed-fluid-cell). In order to measure residual N_2 in experiments where the fluid cell was first purged with Ar, humid Ar gas was transferred through the AFM cell and into a multilayer foil gas transfer bag (VRW international) that had first been purged with Ar and evacuated. The gas from the transfer bag was introduced to a vacuum chamber equipped with a quadrupole mass spectrometer (Extrel QMS). Mass spectra were acquired for the Argon sample and compared to a bag filled with ambient air for reference. In the sample with Argon, the Ar peaks of 20 and 40 amu were dominating as expected. Peaks of 14 and 28 amu (N_2) and 32 amu (O_2) associated with air were rather small, approximately 1% relative to the pure air sample.

KBr/KNO_3 Morphology

Freshly cleaved KBr surfaces exhibit clearly defined step edges and sharp angles at the termination of steps, Fig. S1a. Upon exposure to high humidity (60% RH) step edge termini immediately soften and round. Once this initial reconstruction is complete the surface is quite stable as evidenced by the lack of step movement after 1 day, Fig. S1b, c KBr heated in ambient air results in a recrystallization of KBr forming small crystallites approximately 390 to 590 nm long, 150 to 180 nm wide, and 1.9 to 2.8 nm in height, Fig. S2a. These elongated crystallites adopt an orientation parallel or 90° to major KBr step edges. IR analysis does not show peaks characteristic of KNO_3 after heat treatment. Similar crystallites can be observed after long term irradiation in air or Ar/1% air alongside large KNO_3 crystals, Fig. S2b, c. KNO_3 crystals grown for more than 24 hours, with the gas environment replenished every 5 hours, often adopt an elongated morphology extending up to dozens of microns with heights ranging from 12 to 33 nm growing at ~90° angles and rotated ~45° from the KBr substrate step edges, Fig. S2b. This orientation is consistent with the expected epitaxial relationship between KBr (111) and γ-KNO_3 (003) as measured by XRD, Fig. S2c, S4.

KNO_3 morphology varied greatly even under identical conditions falling within one of four motifs consisting of a triangular (Fig. S3a near, b far), needle-like (Fig. S3a far, b near), branched (Fig. S3c), and rectangular (Fig. S3a far). Although the same general motifs are observed, attempts to replicate the identical morphology under identical experimental conditions were not successful suggesting parameters that were difficult to control (KBr thickness, local

step edge density, etc.) may play a key role. In most cases two to three morphologies were present in each irradiation experiment distributed by proximity to the X-ray source, Fig. S3a-c. Generally, the surface coverage and total KNO_3 volume were greatest nearest to the X-ray source, while the average height of the crystals relative to the KBr substrate increased, Table S1.

KBr/KNO_3 Analysis

Infrared (IR) spectra were collected using a single reflection ZnSe attenuated total reflectance cell (FastIR, Harrick Scientific) mounted in a Bruker Vertex 80v spectrometer equipped with a DTGS detector and a water-cooled Globar source. A spectrum of a KBr crystal irradiated in humid air was collected by pressing the crystal against the ZnSe crystal. A reference spectrum of potassium nitrate (Aldrich) was collected by evaporating a suspension of KNO_3 in isoproponal onto the ATR crystal. All background and sample single-channel spectra were an average of 512 scans at 4 cm^{-1} resolution.

µXRD pole figures confirm the surface as KBr {001} through the expected 4-fold symmetry and 55° inclination of the <111> (Fig. S4a) and 45° inclination of the <220> lattice vectors (Fig. S4b) with respect to the crystal surface. The γ- KNO_3 <003>, <110>, and <202> directions also exhibited 4-fold symmetry and were found inclined at 43°, 53°, and 50° degrees respectively from the KBr surface (Fig. S4c-e). These angles identified the surface of γ-KNO_3 as parallel to the (012) lattice plane. The 4-fold symmetry arises from the four equivalent directions that γ-KNO_3 could be oriented on the cubic KBr {001} surface. The additional maxima in the γKNO_3 <110> figure arise from the 2 directions that this lattice vector makes with the (012) surface giving rise to 8 rather than 4 positions.

The rectangular lattice in γ-KNO_3 is similar to the square KBr lattice and explains why the KBr <111> and γ-KNO_3 <003> directions are aligned in their pole figures. The shorter dimension of the rectangle, 435 pm, is a relatively close match to the K-K distances on the KBr surface (467 pm). The longer dimension, 547 pm, is 17% larger than in KBr and will require mechanisms such as line defects and/or superlattice epitaxy to maintain coherence. This is likely to be responsible for the elongated spots observed in the γ-KNO_3 pole figures. β-KNO_3 contains a similar rectangular lattice of K atoms within (102) but α-KNO_3 has no such arrangement. The formation of γ- KNO_3 over the other polymorphs is therefore likely a combination of epitaxy and the surface stabilization proposed by Scott et al.[22]

NanoSIMS analyses were performed using a NanoSIMS 50L. Samples were embedded in Field's metal and coated with 20 nm high-purity gold prior to analyses. Samples were analyzed using a 16 keV, 1 pA primary Cs^+ beam of ca. 100 nm diameter. $^{12}C^-$, $^{16}O^-$, $^{12}C^{14}N^-$, $^{12}C^{16}O_3^-$, $^{14}N^{16}O_3^-$, and $^{79}Br^-$ ions were accelerated to 8 keV and collected simultaneously using electron multipliers. $^{12}C^{16}O_3^-$ and $^{14}N^{16}O_3^-$ peaks were mass calibrated using $CaCO_3$ and KNO_3 salts respectively. Each image consisting of 8, 256 × 256 pixel, 20 µm × 20 µm frames was deadtime corrected, aligned, and summed using the OpenMIMS plugin for Fiji. Dwell time was 6.75 ms px^{-1} resulting in analysis times of ca. 1 hr. Under these conditions, charging was not observed for two consecutive images (16 total frames) as judged from the secondary electron image and stable signal for secondary ions. Instrumental conditions included a 30 µm entrance slit, D1 of 150 µm, open and centered energy slits, and 100 µm exit slits. The possible ferroelectric and ferromagnetic properties of γ-KNO_3 resulting from irradiation of KBr was analyzed at the local level using piezoresponse force microscopy (PFM) and magnetic force microscopy (MFM). KBr irradiated in 60% RH Ar for 12.4 hrs, with the gas replenished after 5

hours, was aged for 4 months in ambient conditions before analysis. PFM topography shows the formation of large KNO_3 crystals ranging in height from 97 to 296 nm, Fig. S5a. The piezoresponse image shows a clear response of the γ-KNO_3 crystals with a variation in the magnitude of response that corresponds with a shift in the phase of piezoresponse (inset), which denotes differences in domain polarization. Likewise, MFM topography of the same sample shows a large γ-KNO_3 crystal ranging in height from 87 to 142 nm, Fig. S5b. There is a clear magnetic response caused by a shift in amplitude during the second pass of the tip 500 nm above the sample. These results are strong indicators of the presence of ferroelectric/magnetic properties expected of gamma phase KNO_3.

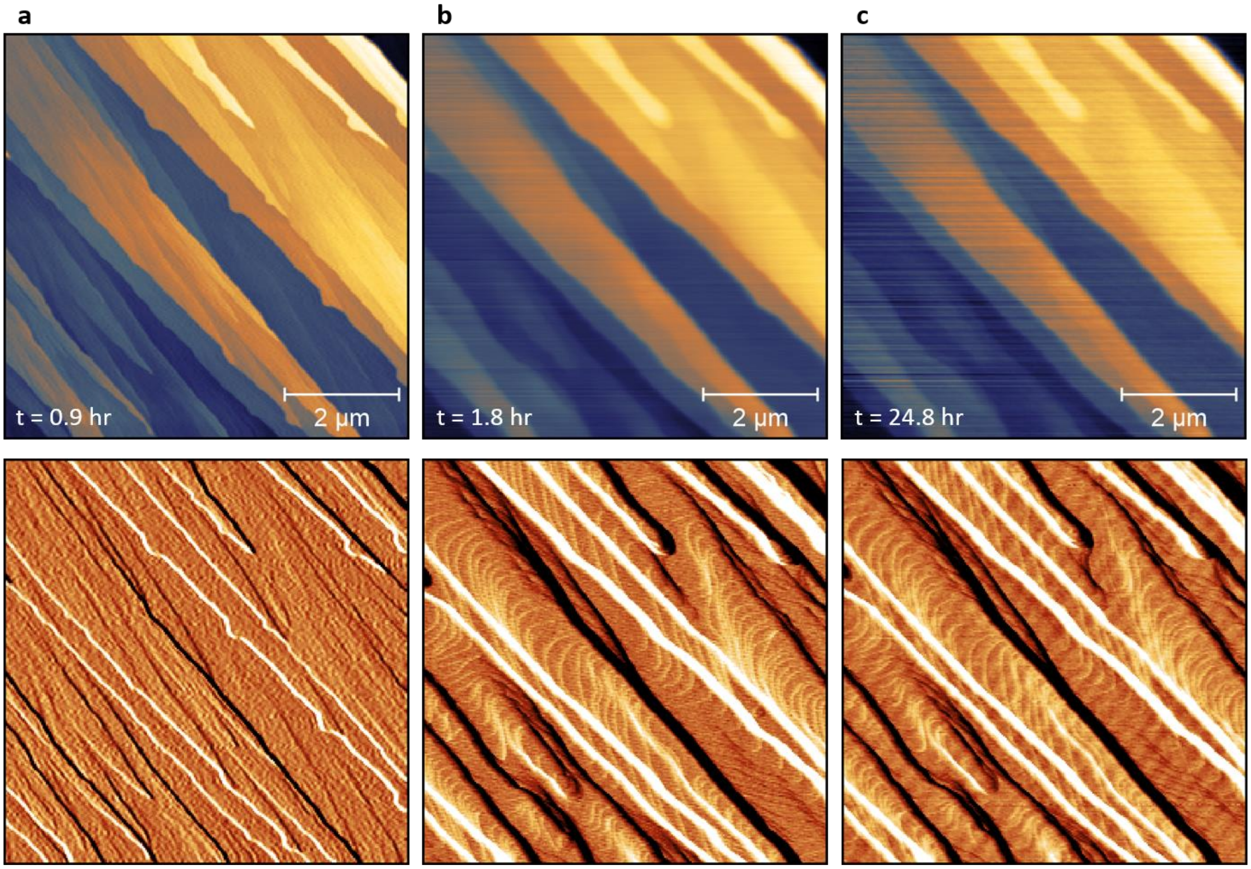

Fig. S1. KBr surface reconstruction after cleaving. AFM topography (top) and amplitude (bottom) of a KBr single crystal 0.9 hrs after cleaving (a), 0.9 hours after flowing 60 % RH Ar (b), and 23.9 hours after introducing humid Ar (c).

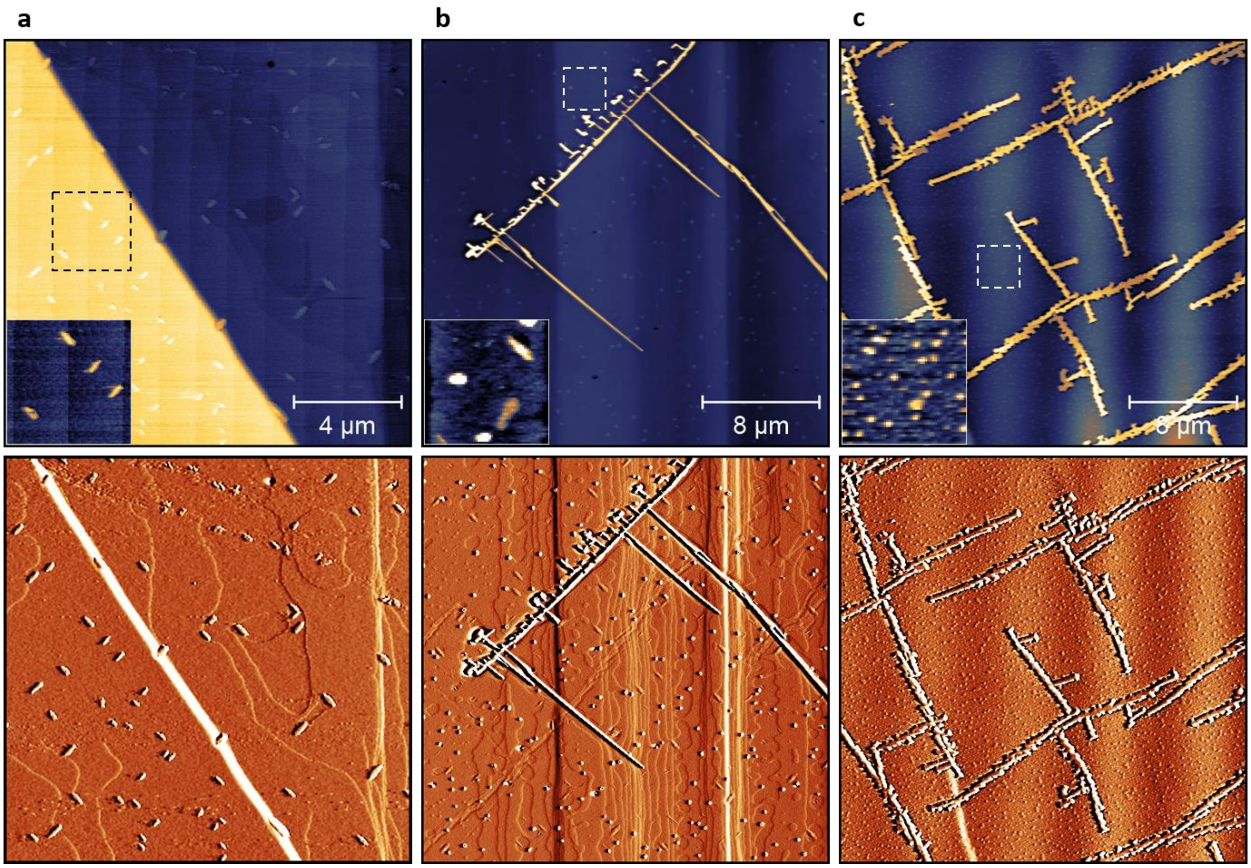

Fig. S2. KBr crystallite growths after heating and long-term irradiation. Contact mode AFM topography (top) and deflection (bottom) of crystallites formed on the KBr surface after heating in air at 50°C for 20 hours (a), after long-term irradiation in 60% RH air for 36.7 hours (b), and after long-term irradiation 60 % RH Ar/ 1% air mix for 44.4 hours (c). Inset images are 3 µm x 3 µm.

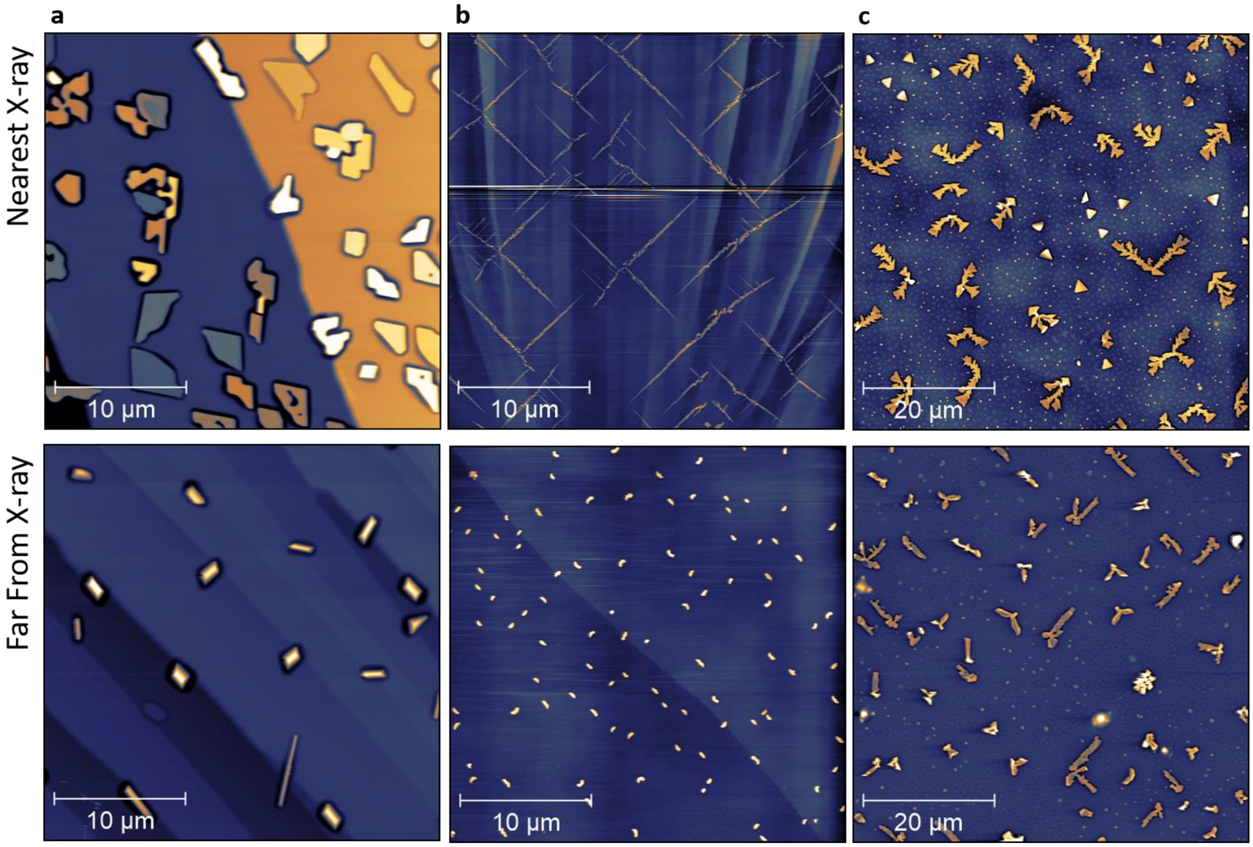

Fig. S3. KNO_3 crystal morphology is dependent on proximity to the X-ray. AFM topography of KBr irradiated for 22.4 hrs in 60% RH air (a), 15.0 hrs in 60% RH air (b) and 21.6 hrs in 60 % RH Ar/1% air mix (c) display the variety of KNO_3 morphologies observed and the impact of X-ray proximity.

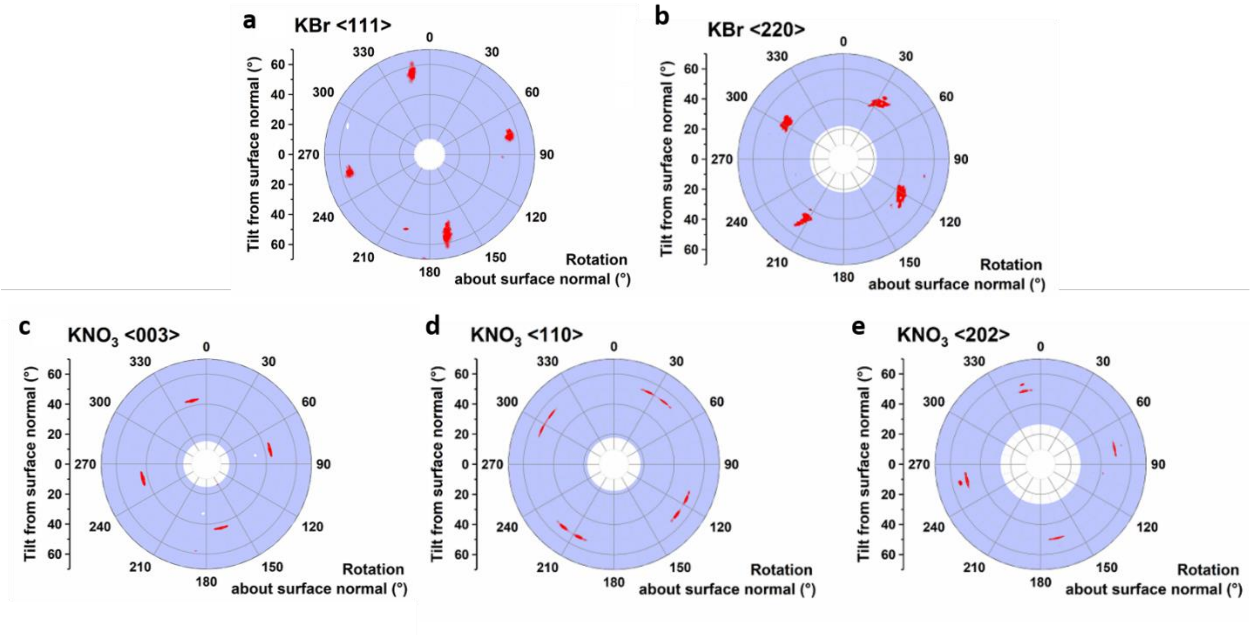

Fig. S4. Pole figures showing the orientations of (a) KBr <111> and (b) KNO_3 <003> lattice vectors. These are consistent with KBr (001) and KNO_3 (012) surfaces. Pole figures identify the surface plane of γ-KNO_3 (c)-(e).

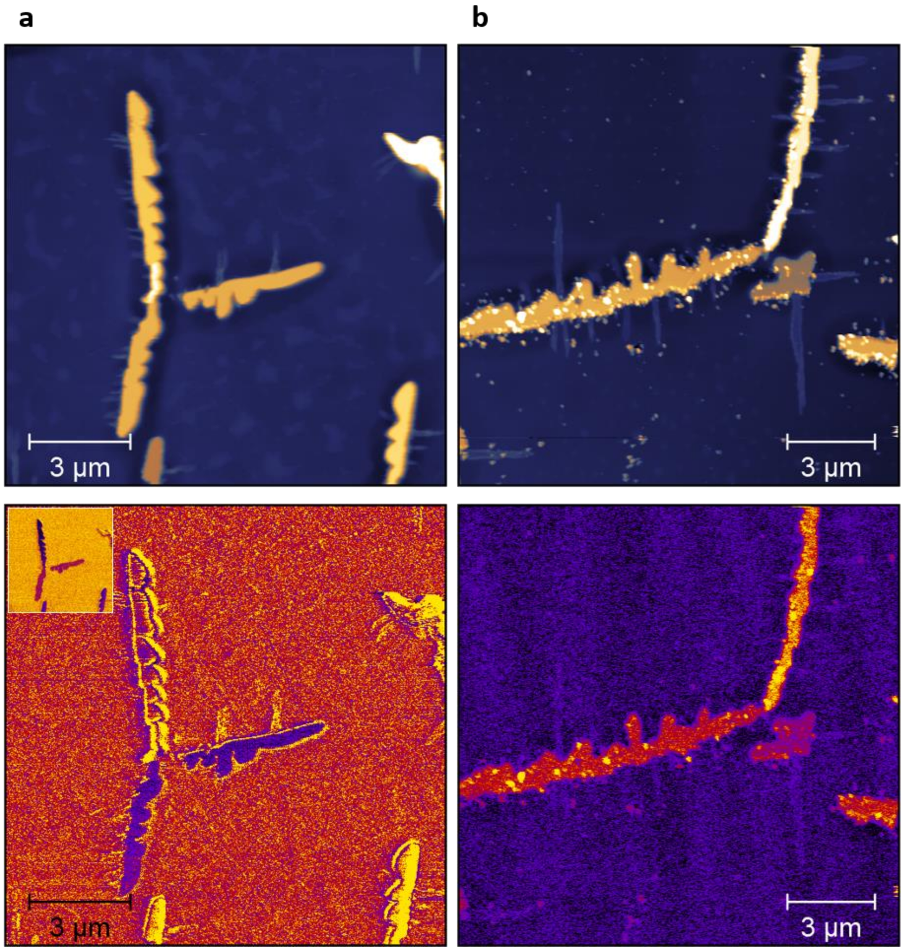

Fig. S5. AFM measurement of the local ferroelectric and ferromagnetic response of γKNO_3 grown on irradiated KBr. PFM topography, piezoresponse, and piezoresponse phase (inset) of γ-KNO_3 (a). MFM topography and magnetic response (amplitude) of γ-KNO_3 (b).

	(a) near	(a) far	(b) near	(b) far	(c) near	(c) far
Total X-ray dose to cell (kGy)	400	400	270	270	390	390
% Surface Coverage	17.2	2.6	5.8	2.0	7.3	5.9
Average Height (nm)	46.7	285.7	18.1	35.9	51.0	55.5
Vol. of KNO_3 per area ($\mu m^3/\mu m^2$)	$8.0x10^{-3}$	$7.4x10^{-3}$	$1.1x10^{-3}$	$7.1x10^{-4}$	$3.7x10^{-3}$	$3.3x10^{-3}$

Table S1. Comparison of KNO_3 growth as a function of X-ray proximity. Analysis of AFM topography presented in Fig. S3 show KNO_3 surface coverage and volume increase near the X-ray source, while height decreases.
