# Supplementary material for: Direct visualization of radiation-induced transformations at alkali halide–air interfaces
Source: Commun Chem. 2021 Apr 8;4:49. doi: 10.1038/s42004-021-00486-2 (PMC9814822; doi:10.1038/s42004-021-00486-2)
Supplement: Supplementary file 2 — Description of Additional Supplementary Files [file 42004_2021_486_MOESM2_ESM.pdf]

Description of Additional Supplementary Files

File Name: Supplementary Movie 1-3
Description: Time lapse AFM topography, amplitude, and phase respectively of KBr irradiated in 0.1 % RH air over 12 hours as shown in Figs. 3a-b, 4a.

File Name: Supplementary Movie 4-6.
Description: Time lapse AFM topography, amplitude, and phase respectively of KBr irradiated in 60 % RH Ar/1% air over 8 hours as shown in Figs. 3c-d, 4b.
