# Supplementary figures and images for: Direct visualization of radiation-induced transformations at alkali halide–air interfaces
Source: Commun Chem. 2021 Apr 8;4:49. doi: 10.1038/s42004-021-00486-2 (PMC9814822; doi:10.1038/s42004-021-00486-2)

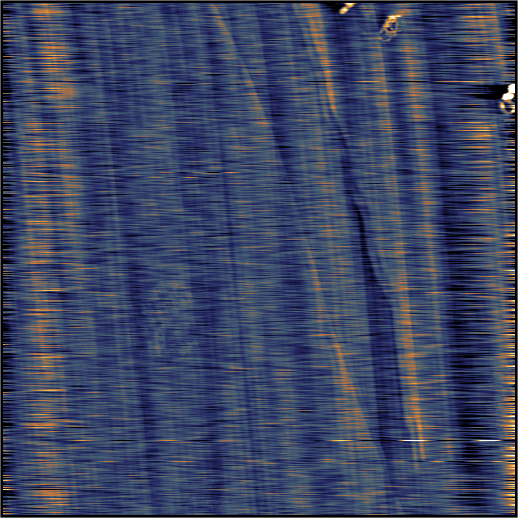

Supplement: Supplementary file 3 — Supplementary Movie 1 [file 42004_2021_486_MOESM3_ESM.gif]

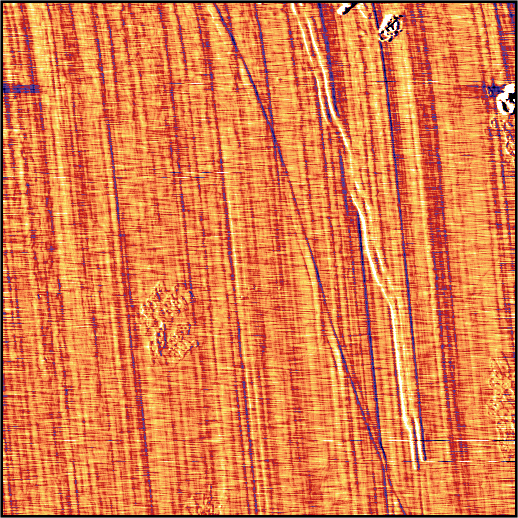

Supplement: Supplementary file 4 — Supplementary Movie 2 [file 42004_2021_486_MOESM4_ESM.gif]

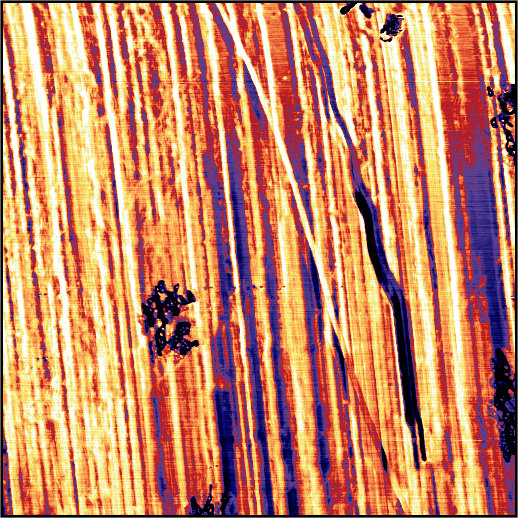

Supplement: Supplementary file 5 — Supplementary Movie 3 [file 42004_2021_486_MOESM5_ESM.gif]

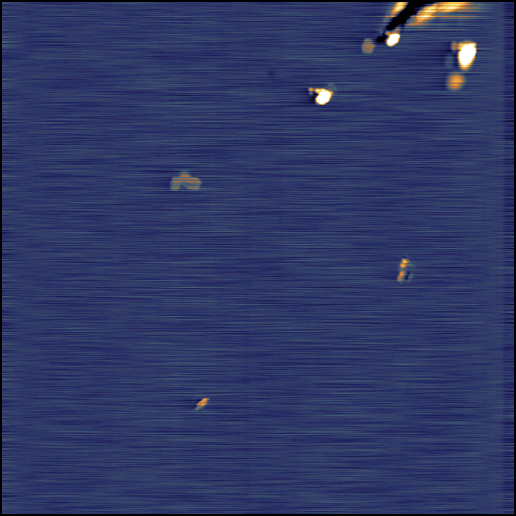

Supplement: Supplementary file 6 — Supplementary Movie 4 [file 42004_2021_486_MOESM6_ESM.gif]

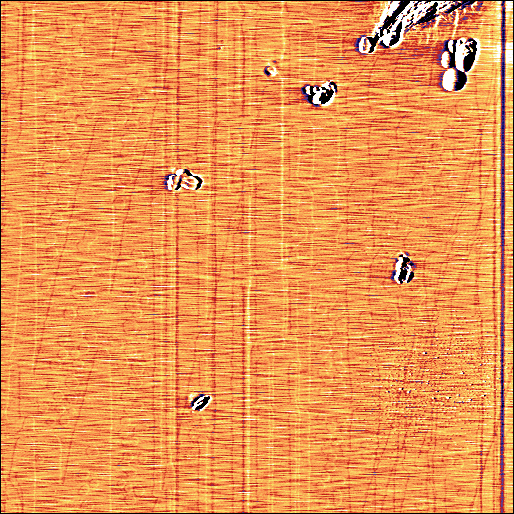

Supplement: Supplementary file 7 — Supplementary Movie 5 [file 42004_2021_486_MOESM7_ESM.gif]

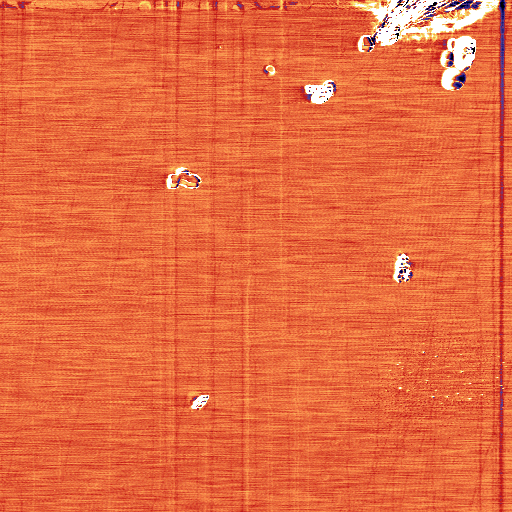

Supplement: Supplementary file 8 — Supplementary Movie 6 [file 42004_2021_486_MOESM8_ESM.gif]
